# Prevalence of Food Insecurity and Its Associated Factors among Adult People with Human Immunodeficiency Virus in Ethiopia: A Systematic Review and Meta-Analysis

**DOI:** 10.1155/2021/7816872

**Published:** 2021-06-01

**Authors:** Hagos Degefa Hidru, Haftay Gebremedhine, Alem Gebretsadik, Hirut Teame, Hadush Negash, Meresa Berwo Mengesha

**Affiliations:** ^1^College of Medicine and Health Sciences, Department of Public Health, Adigrat University, Adigrat, Ethiopia; ^2^College of Medicine and Health Sciences, Department of Laboratory Unit of Medical Microbiology, Adigrat University, Adigrat, Ethiopia; ^3^College of Medicine and Health Sciences, Department of Midwifery, Adigrat University, Adigrat, Ethiopia

## Abstract

**Background:**

Food insecurity is the shortage of both the quantity and quality of food and a negative impact on the overall nutritional and health status of people with human immunodeficiency virus (HIV). Ethiopia is intensely affected by food insecurity which is about 87.4% of adult people living with human immuno deficiency virus (HIV) are still facing shortage to have access to safe, sufficient, and nutritious food for themselves and their family. However, there is no concrete scientific evidence established at the national level in Ethiopia. Hence, this review gave special emphasis on adult people with human immunodeficiency virus (HIV) to estimate the pooled prevalence of food insecurity and its associated factor at the national level in Ethiopia.

**Methods:**

Studies were retrieved from selected electronic data bases, including PubMed/Medlin, Cochrane library, Sciences Direct, Google, and Google Scholar. Random-effects model meta-analysis was used to estimate the pooled prevalence of food insecurity and its associated factors at 95% confidence interval with odds ratio (OR) using statistical *R*-software version 3.6.1. Moreover, quality appraisal of the included studies, publication bias was checked using the funnel symmetry test, and heterogeneity was checked using forest plot and inverse variance square (*I*^2^). The searches were restricted to articles published in the English language only, and Medical Subject Headings (MeSH terms) was used to help expand the search in advanced PubMed search.

**Result:**

A total of 650 articles were identified through the initial search of which 20 studies were included in the final review yielding a total sample size of 7,797 adult people with human immunodeficiency virus (HIV). The pooled prevalence of food insecurity was 52% (95% CI, 40%, 63%). Cluster of differentiation 4 (CD4) count < 350 cell/mm^3^ [AOR = 1.29 (95% CI, 1.08, 1.54)], develop opportunistic infection [AOR = 4.09 (95% CI, 2.47, 6.78)], rural residence [AOR = 1.59 (95% CI, 1.09, 2.34)], and World Health Organization (WHO) clinical stages III and IV [AOR = 1.98 (95% CI, 1.23, 3.19)] was among the significantly associated factors.

**Conclusion:**

In this review, there was a high prevalence of food insecurity among adult people with human immunodeficiency virus. Therefore, the responsible stockholders should strengthen the system and procedure for early diagnosis of opportunistic infection, under nutrition, screening of underlying problems.

## 1. Introduction

The World Bank and United Nations define food security as people having at all times physical, social, and economic access to sufficient, safe, and nutritious food which meets their dietary needs and food preferences for an active and healthy life [1–5]. Food insecurity defined as lack of access to sufficient, safe, nutritionally adequate foods or needing to acquire foods to meet dietary needs and maintain a healthy and active life [6–8].

Food insecurity is the shortage of both quantity and quality food [9, 10]. Due to the emerging global changes in social, economic, political, and climatic conditions, food insecurity is becoming a focus of public health and political attention in low- and middle-income countries, particularly in sub-Saharan Africa. The number of people experiencing food insecurity is expected to double by the year 2020 [11, 12]. Human immunodeficiency virus (HIV) epidemic largely overlaps with populations already experiencing low diet quality and quantity [6, 13]. Food insecurity and people with HIV infection have a direct relationship as malnutrition increases susceptibility to the HIV infection [8].

Food insecurity has a negative impact on the overall nutritional and health status for those people with HIV and often expresses that food is the greatest need for themselves and their families [14, 15]. Food insecurity, in turn, leads to both increased risk of HIV transmission and more rapid HIV disease progression across settings in both resource-rich and resource-poor countries [10]. Combating undernutrition and HIV/AIDS was 2 of the 8 United Nations Millennium Development Goals to be achieved by 2015 international targets that form a blueprint for galvanizing priorities for the world's poor, but still they are the most challenges in the sub-Saharan country [6].

According to FAO, 2012-14 report, globally, about 805 million, 791 million people in developing countries estimated to be chronically hunger, and the sub-Saharan Africa region remains the highest prevalence in about one in four of their people which are food insecure [16–20]. The five African countries with the most number of people in state of hunger have 10 and 32 million people each in state of food insecure to take adequate food, and four of them are in the horn of Africa (Ethiopia 32.1 million, Tanzania 15.7 million, Kenya 11 million, and Uganda 10.7 million) [21–24]. Globally, an estimated 34 million adult people were living with HIV/AIDS with 2.7 million new infections. Sub-Saharan Africa also home to approximately 24·7 million people living with HIV which accounting for about 70% of the global total of new people with HIV and 1.8 million people dying annually from AIDS-related causes [25–28]. Studies done in Africa showed that about 52.2% to 92% of the people living with HIV/AIDS are struggling to have access to safe and nutritious food for themselves and their families. Ethiopia is one of many sub-Saharan African countries intensely affected by food insecurity, and about 10% of the general population and 40.4% to 87.4% adult people living with HIV/AIDS will struggle to have access to “safe, sufficient and nutritious food” for themselves and for their families [29–33].

The prevalence of food insecurity is inconsistent across regions and had variations over time among different single studies. Similar to the prevalence of food insecurity, predictor variables were also inconsistent among the studies. In addition to this gap, lack of documented and published peer-reviewed data on the pooled prevalence of food insecurity and its associated factors among adult people with HIV at the national level in Ethiopia hinders program managers to design and implement effective strategies. With there is minimal existing evidence in developing countries, including Ethiopia, evidence-based on pooled results at the national level on prevalence and factors of food insecurity among adult people with HIV is needed to guide decision-making and implementation programs. Therefore, this review would pool the evidence of food insecurity and its factors among adult people with HIV at the national level of Ethiopia.

## 2. Methods

### 2.1. Searching Strategies

We systematically reviewed and analyzed published research articles to determine the pooled prevalence of food insecurity and its associated factors among adult people with HIV in Ethiopia. We identified and searched published articles using major electronic databases, including PubMed/Medline, Cochrane library, Google and Google Scholar, and Sciences Direct, as well as a manual search from grey literature that were conducted accordingly. The key terms/phrases of Medical subject Headings (MeSH) used for PubMed search were “Prevalence” AND “Food” OR “Nutrition” OR “Diversity” AND “Insecurity” OR “Inadequate” AND “HIV” OR “ADIS” AND “Ethiopia.” The searches were restricted to articles published in the English language only. The search terms were predefined to allow a comprehensive search strategy that included all fields within records, and Medical Subject Headings (MeSH terms) was used to help expand the search in advanced PubMed search. This study also used Boolean operator (we combined keywords with the “OR” operator, and we then linked the search strategies with the “AND” operator).

#### 2.1.1. Eligibility Criteria

We reviewed articles from the initial search using defined inclusion and exclusion criteria.

*(1) Inclusion Criteria*. Community and institutional-based cross-sectional studies published in the English language from 2012 until 2018 irrespective of their sample size which focus on the prevalence of food insecurity and its associated factors among adult people with HIV/AIDS at the national level in Ethiopia were included in this systematic review and meta-analysis.

*(2) Exclusion Criteria*. Studies that did not report specific outcomes either for the food insecurity or associated factors or both quantitatively were excluded from this systematic review and meta-analysis. Those papers which could not be fully accessed at the time of our search process were excluded from this review after a contact was attempted with the principal investigator through email on two occasions. Generally, articles not related to our review were excluded during title, abstract, and full-text screening.

### 2.2. Data Extraction and Synthesis

The database search results were combined, and duplicate articles were removed using Endnote (versionX8). Data were extracted by two authors using a standardized data extraction spreadsheet. Data extraction spreadsheet was pretested on five randomly selected articles, and modified was done accordingly. The spreadsheet included study characteristics such as (1) authors' name, year of study, regions, publication year, total sample size, study design, and study population; (2) frequency of food insecurity and security; and (3) data also extracted for factors presented for each respective studies with their frequency of food secured and insecure included sex, residence, CD4count, WHO clinical stage, hemoglobin level, and opportunistic infections. The qualities of all the included studies were substantiated using a quality score from the Joanna Briggs Institute (JBI).

The initial screening of the articles by title, abstract, and full text was carried out by two authors (HDH and MM) independently based on the predefined inclusion and exclusion criteria. After each screening round (title, abstract, and full texts), the authors met and resolved any discrepancy by discussion, while potential disagreements were solved by the involvement of the rest of the authors (HG, AG, HT, and HN). The reference lists of the included full-text articles were appraised to ascertain additional articles of relevance to determine necessity of retrieving the full text. Finally, all the included accessible full-text articles were extracted to assemble appropriate information based on the inclusion criteria.

### 2.3. Quality Assessment

The quality of all the included articles was assessed for their risk bias using Joanna Briggs Institute Meta-Analysis of Statistics Assessment and Review Instrument (JBI-MAStARI) adapted for cross-sectional and cohort study design [34]. Two independent reviewers critically appraised each paper. Disagreements between those reviewers were solved by discussion. Studies which scored between five and nine were included in the final systematic review and meta-analysis.

#### 2.3.1. Outcome Measurement

The primary aim was to know the pooled prevalence of food insecurity among adult people with HIV/AIDS at the national level, while the secondary aim of this review was to identify different factors affecting food insecurity among adult people with HIV in Ethiopia. By aggregating these two-level information, it will build understanding for future implemented for a policy intervention program in order to decrease the high burden of food insecurity and HIV-related complication.

#### 2.3.2. Data Synthesis and Analysis

Prevalence of food insecurity and estimates for risk factors obtained from each study were pooled and determined as a single estimate. The extracted data were entered into the computer via an Excel sheet for screening their title, abstract, and full texts and then exported to *R*-statistical software version 3.6.1 for analysis. Publication bias was assessed using the funnel symmetry test and sensitivity test. The heterogeneity test across studies was done using the inverse variance square (*I*^2^) with the Cochrane *Q* statistic test. Forest plots to visualize heterogeneity for the pooled prevalence using the random-effects model were also conducted. Risk factors obtained from each primary study were thematically organized, and their effect sizes were pooled accordingly using the random-effects model. The Preferred Reporting Items for Systematic Reviews and Meta-Analysis (PRISMA) statement for reporting a systematic review and meta-analysis was used to clearly present the study inclusion, exclusion, and rationales for exclusion information in the diagram as follows under the results section.

## 3. Results

**Figure 1:**
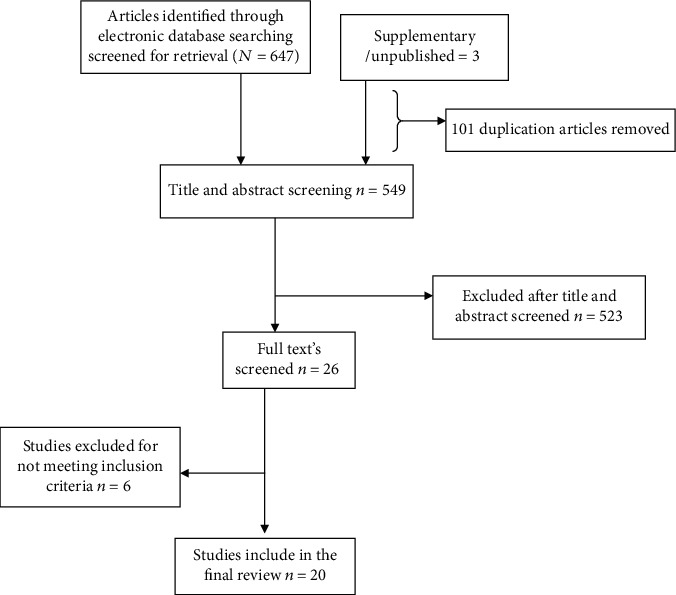
Flowchart shows selected and screening of articles for systematic review and meta-analysis.

In this review, we extracted data related to food insecurity and their predictors among adult people with HIV at the national level. A total of 650 articles were retrieved from different sources through the electronic database (647) and supplementary from unpublished (3) searches of which 101 duplicated articles were excluded after screened had done. From the remaining 549 articles, 523 articles were excluded after reading of their titles and abstracts based on the predefined inclusion criteria. Finally, 26 full-text articles were accessed and assessed for the eligibility criteria. Based on the predefined criteria and after critical appraisal, only 20 articles were included for the final analysis. In case of significant heterogeneity using the inverse variance square (*I*^2^) in the random effect model along with 95% confidence interval, we did subgroup analysis using sampling technique, study design (institutional and community-based cross-sectional), publication year, sample size, and regions, and no changed value on the heterogeneity was observed except for the sample size variation (Figure 1).

### 3.1. Characteristics of Included Studies

A total of twenty articles had met the inclusion criteria. All the included studies were published between 2012 and 2018 in the English language. Twenty cross-sectional studies were included using an estimated sample size range from 109 [16] up to 630 [8] adult people with HIV samples who were taken at Oromia parts of Ethiopia in the year 2016 and Addis Ababa city in the year 2014, respectively. A total sample of 7,797 adult people with HIV were included to estimate the pooled prevalence of food insecurity and its associated factors among adult people with HIV at the national level in Ethiopia (Table 1). Of the total 20 articles, near to half of studies were conducted in South Nations Nationalities and Peoples of Ethiopia (SNNP), seven studies in the Oromia regional state, three studies in the Amahara regional state, and one study at the Tigray national regional state and in Adis Abeba specialized hospital (Table 1**).**

### 3.2. Prevalence of Food Insecurity among Adult People with HIV in Ethiopia

**Figure 2:**
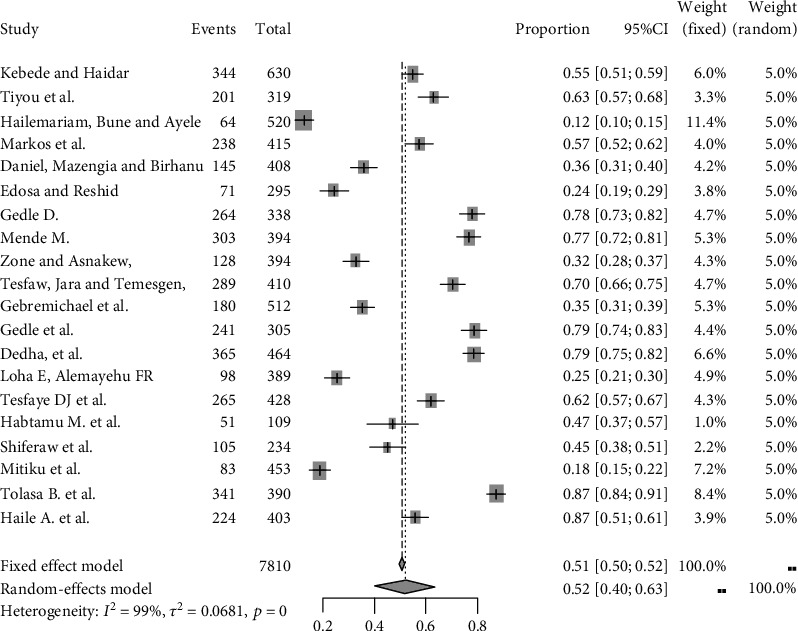
Forest plot showing the pooled prevalence of food insecurity among adult people with HIV in Ethiopia from 2012 up to 2018.

The pooled prevalence of food insecurity among adult people with HIV at the national level in Ethiopia was found to be 52% (95% CI, 40%, 63%), using visual forest plots in the random-effects model along with 95% confidence interval (Figure 2).

### 3.3. Associated Factors of Food Insecurity among HIV-Infected Adult Individuals in Ethiopia

**Figure 3:**
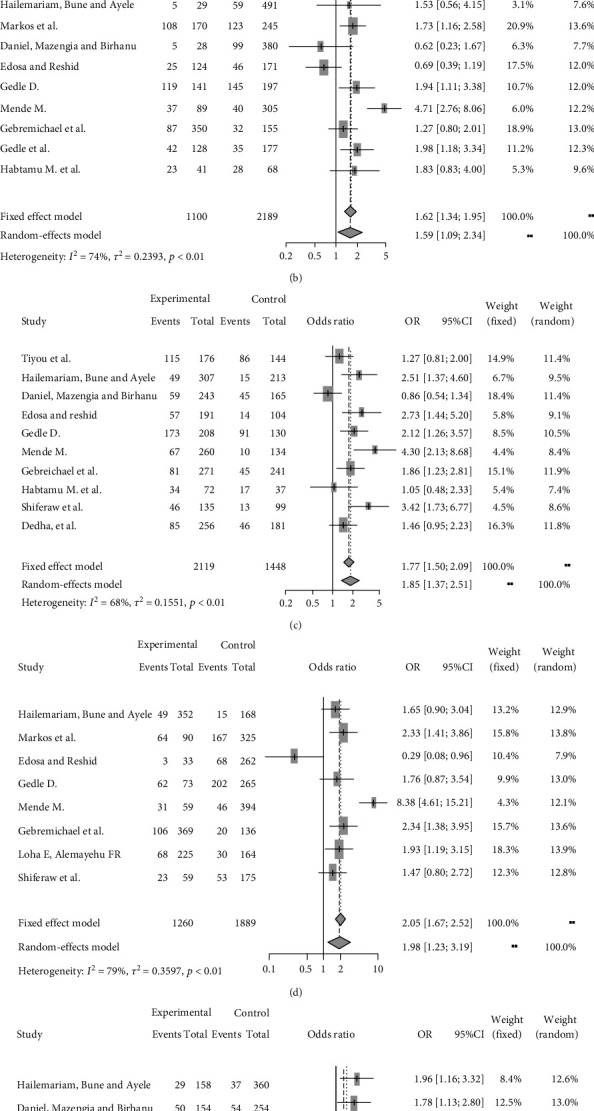
(a) Forest plot which shows the association between food insecurity and having opportunistic infection among adult people with HIV in Ethiopia from 2012 up to 2018. (b) Forest plot which shows the association between food insecurity and rural residence among adult people with HIV in Ethiopia from 2012 up to 2018. (c) Forest plot which shows the association between food insecurity and being female in gender among adult people with HIV in Ethiopia from 2012 up to 2018. (d) Forest plot which shows the association between food insecurity and WHO clinical stages III and IV among adult people with HIV in Ethiopia from 2012 up to 2018. (e) Forest plot which shows the association between food insecurity and CD4 count among adult people with HIV in Ethiopia from 2012 up to 2018.

Low CD4 counts, WHO clinical stage, opportunistic infection, rural residence, and being female in gender were found to have a significant association with the occurrence of food insecurity among adult people with HIV at the national level. Presence of opportunistic infection was 4.09 times more associated with food insecurity among people with HIV as compared people with HIV who are free from opportunistic infection [AOR = 4.09, (95% CI, 2.47, 6.78)] (Figure 3(a)). Rural residence was 1.59 times more associated with food insecurity among people with HIV as compared with those people with HIV who resides in urban [AOR = 1.59 (95% CI, 1.09, 2.34)] (Figure 3(b)). Female with HIV were 1.85 times associated with food insecurity as compared to male with HIV [AOR = 1.85 (95% CI, 1.37, 2.51)] (Figure 3(c)). WHO clinical stages III and IV were 1.98 times more associated with food insecurity as compared to those people with HIV clinical stage of I and II [AOR = 1.98 (95% CI, 1.23, 3.19)] (Figure 3(d)). Their CD4 count < 350 cell/mm^3^ of people with HIV was 1.29 more associated with food insecurity as compared to people with HIV which their CD4 count were ≥350 cell/mm^3^ [AOR = 1.29 (95% CI, 1.08, 1.54)] (Figure 3(e)).

## 4. Discussion

This systematic review and meta-analysis were established out to estimate the pooled prevalence of food insecurity and its associated factor among adult people with HIV at the national level in Ethiopia from 2012 to 2018. According to this review, more than half of adult people with HIV/AIDS were found to be food insecure in Ethiopia. The prevalence of food insecurity in the current systematic review and meta-analysis was found to be 52% from the random-effects model, which is higher than the study conducted in West Bengal (49%) [35]. This difference may be due to variation in the sample size of the study and on the measurement cut point to say food insecure, difference in study period, and other differences may be due to attributable geographical location variation between these countries: immunity status, presence of opportunities infections, and severity/clinical stage of HIV/AIDS.

The finding of this study was also lower than the finding of the study conducted in Nigeria (71.7%) [11], Canada 73%) [36], South Africa (60%) [37], and Ugandan (78.5%) [38]. These variations might be due to difference in the sample size, measurement items of food insecurity, and the cut point to say food secure/insecure from country to country, difference in the study period (seasonal difference), difference on the commonly utilized, available food in their place of residence, geographical location variation, and culturally and/or religiously aspects of foods as well knowledge differences on ways of access to information on different food items, educational level, economical status, family size, and food security status.

## 5. Theoretical and Practical Implications

People with HIV/AIDS are more prone to develop concomitant infection and easily affected by food insecurity. The evidence of this review also indicated that those food insecure people with HIV deserved more attention where integrated service provision and manage underlying cause at early stages. Hence, specific considerations tailored to food insecurity screening and management need to be taken into account during ART program design and implementation across the health system. Despite the vast investment of resources in tackling the occurrence of food insecurity in low- and middle-income countries, few individual studies are available to inform policy and decision makers. Specifically, food insecurity can lead to macronutrient and micronutrient deficiencies, which can affect both vertical and horizontal transmission of HIV, and can also contribute to immunologic decline and increased morbidity and mortality among those already infected. Probability of AIDS is defining illness and AIDS-related mortality among people with HIV [28]. Therefore, further studies are also recommended to generate pooled evidence based at global in general and in developing countries in particular.

## 6. Strength and Limitation

The main strength of the current review lies in our adherence to the international standardized guidelines on the conduct and reporting of systematic review and meta-analysis at the national level. We include studies only from peer-reviewed English language journals, which may have restricted our findings. Though searching was done in analytical studies (cohort and case-control studies), only cross-sectional study design was included because we did not find any published case-control and cohort-related studies through the searching engine we had used.

## 7. Conclusion

This systematic review and meta-analysis showed a high prevalence of food insecurity among people with HIV at the national level in Ethiopia in which more than half of people with HIV/AIDS were food insecure. The review had found that factors like low CD4 count (<350cell/mm^3^), WHO clinical stages III and IV, presence of opportunistic infection, being female in gender, and rural residence were among the significant associated factors with the development of food insecurity among adult people with HIV/AIDS. Therefore, the responsible stockholders including ART clinics should strengthen the system and procedures for the early diagnosis of opportunistic infection. In order to alleviate the food security problem among adult people with HIV at the national level in Ethiopia, the government should coordinate and integrated food assistance and HIV program at all levels. Create linkage and early screened between community-based nutrition interventions for PLWHA and likelihood support and food assistance intervention. Moreover, the system should focus on technical update training on opportunistic infection diagnosis and treatment guidelines, on nutritional assessment counseling and support (NACs), and strengthening of laboratory facilities that can detect opportunistic infections early in infection. There should also be strict and frequent monitoring of people with HIV than the previous and should work in collaboration with agricultural sectors especially for the rural settings in order to avail themselves on food access in terms of quality and quantity to minimize food insecurity burdens.

## Figures and Tables

**Table 1:** Characteristics of included studies to estimate pooled prevalence of food insecurity and its predictors among HIV-infected adult individuals in Ethiopia from 2012 up to 2018.

Id	Region	Authors (reference)	Publication year	Study design	Total sample size	Frequency of food		Quality score
						Secure	Insecure	
1	SNNP	Gedle et al. [17]	2015	Cross-sectional	305	64	241	5
2	SNNP	Mende [18]	2017	Cross-sectional	394	91	303	6
3	SNNP	Gedle [19]	2017	Cross-sectional	338	74	264	6
4	SNNP	Zone and Asnakew [20]	2015	Cross-sectional	394	266	128	5
5	Amahara	Tesfawa et al. [21]	2018	Cross-sectional	410	121	289	7
6	Oromia	Tolesa et al. [22]	2015	Cross-sectional	390	49	341	6
7	Adis Abeba	Kebede and Haidar [8]	2014	Cross-sectional	630	286	344	7
8	Oromia	Tiyou et al. [23]	2012	Cross-sectional	319	118	201	5
9	SNNP	Hailemariam, Bune, and Ayele [24]	2013	Cross-sectional	520	456	64	7
10	SNNP	Markos et al. [25]	2018	Cross-sectional	415	177	238	6
11	Amahara	Daniel, Mazengia, and Birhanu [26]	2013	Cross-sectional	408	263	145	6
12	Oromia	Edosa and Reshid [27]	2017	Cross-sectional	295	224	71	5
13	Oromia	Gebremichael et al. [28]	2018	Cross-sectional	512	332	180	7
14	Oromia	Dedha et al. [29]	2017	Cross-sectional	464	99	365	8
15	Amhara	Haile et al. [30]	2015	Cross-sectional	403	179	224	8
16	SNNP	Loha and Alemayehu [31]	2017	Cross-sectional	389	291	98	7
17	SNNP	Tesfaye et al. [32]	2018	Cross-sectional	428	163	265	8
18	Oromia	Habtamu et al. [16]	2016	Cross-sectional	109	58	51	5
19	Tigray	Shiferaw et al. [33]	2017	Cross-sectional	234	129	105	5
20	Amahara	Mitiku et al. [35]	2016	Cross-sectional	453		83	7

SNNP: Southern Nation Nationality People.

## Data Availability

The datasets analyzed during the current study are available from the corresponding author upon reasonable request.

## Abbreviations

AOR: Adjust odds ratio
AIDS: Acquired immune deficiency syndrome
CD4: Cluster of differentiation 4
CI: Confidence interval
FAO: Food and agricultural organization
HIV: Human immunovirus
JBI: Joanna Briggs Institute
MeSH: Medical Subject Headings
PRISMA: Preferred Reporting Items for Systematic Review and Meta-Analysis
SNNP: South Nations Nationalities and Peoples
WHO: World Health Organization.
